# Supplementary material for: Segmental motor recovery after cervical spinal cord injury relates to density and integrity of corticospinal tract projections
Source: Nat Commun. 2023 Feb 9;14:723. doi: 10.1038/s41467-023-36390-7 (PMC9911610; doi:10.1038/s41467-023-36390-7)
Supplement: Supplementary file 1 — Supplementary Information [file 41467_2023_36390_MOESM1_ESM.pdf]

Segmental motor recovery after cervical spinal cord injury relates to density and integrity of corticospinal tract projections

Supplementary Material

Limitations, advantages, and future directions of research

Some limitations, advantages, and future directions of research are explored in this section.

Segmental distribution of motoneurons innervating upper limb muscles

The segmental distribution of motoneurons innervating upper limb muscles is one limitation when using the motor component of the ISNCSCI rating - which is not comparable between segments (as they are non-linear in intervals).[1] For example, the α-motoneurons innervating the elbow flexors (*biceps brachii*) are located at several spinal cord segments (C5-C8), compared to hand muscles (constrained to C8-T1). Also, the motor deficits at different levels of the motor component of the ISNCSCI are not comparable and not necessarily related purely to voluntary motor control nor function.

Selection bias and limitations of the neurophysiological assessments

The calculated segmental percentages of recovery may differ due to selection bias, *i.e.*, the prevalence of C4 and C5 lesions, and with the initial degree of segmental paralysis. Thus, it is more likely that proximal muscles are more affected by lower motor neuron (LMN) lesions, compared to hand muscles. The inability of our machine learning models in predicting proximal muscles that do not recover strength (Figure 5G) may be related to the presence of LMN lesions in muscles with absent recovery. Future studies should investigate if the assessment of other nerves, *e.g.*, the musculocutaneous nerve, could improve the prediction of proximal muscles that do not regain strength after SCI. We also pinpoint limitations of anatomical nature, such as not accounting for the role of other spinal tracts.[2–5] Given the complexity of the cervical spinal neuroanatomy it is important to consider the role of other spinal tracts, *e.g.*, the concept of medial (bilateral, indirect/proprio- and reticulo-spinal) versus lateral (unilateral CST) motor systems in controlling proximal versus distal upper limb muscles, respectively.[2–4] These spinal tracts are neuroanatomically different in terms of grey matter and somatotopy, indicating that projections contralateral/dorsolateral to distal limb α-motoneurons are less redundant than bilateral ventromedial spinal projections to proximal/trunk α-motoneurons. For example, in SCI, it is known that the reticulospinal tract assists hand control during gross finger manipulations.[5]

Another aspect to take into account is the number of α-motoneurons innervating upper limb muscles. It is known from non-human primate experiments that the number of α-motoneurons projecting to proximal upper limb muscles is greater (*biceps brachii* ≈ 1,020; *triceps brachii* ≈ 1,293; *extensor carpi radialis* and *ulnaris* ≈ 1,022) compared to distal hand muscles (intrinsic hand muscles: *flexor pollicis* and *abductor pollicis brevis* ≈ 122; *lateral lumbricalis* ≈ 68; *first dorsal interossei* ≈ 184).[6] Thus, a lesion of similar size may have more pronounced effects on distal hand muscles, compared to proximal upper limb muscles. In this line of thought, this study contributes to a deeper

understanding of the motor impairment and recovery at the segmental, not person, level. The use of the segmental analysis described here will circumvent current difficulties in characterizing the motor impairment at the person level (summed UEMS) by affording the description of the specific motor impairment at each muscle, *e.g.*, muscles with flaccid paralysis (LMN lesion) or lacking control or with spasticity (UMN lesion).

Final considerations on "why it is so hard to regain strength and predict the strength recovery in distal hand muscles?"

The stronger monosynaptic (fast) response of distal forelimb muscles in relation to proximal upper limb muscles also supports the reliance of distal muscles on direct CST projections.[7,8] Around 1,600 motor axons innervate the elbow flexors (*i.e.* main branch of the musculocutaneous nerve), while a similar number of motor axons (about 1,700) are required for the higher number of muscles in all of the distal hand muscles (*i.e.,* ulnar and median nerves at the arm level).[9] This evidence suggests that a similar number of motor units innervate fewer muscles in the proximal arm muscles, compared to distal hand muscles. Thereby, proximal upper limb muscles display lesser direct CST projections[8] and a greater proportion of motor axons per muscle[9], which allows for more redundancy and compensation when the control of some motor units is lost after SCI. For example, the elbow flexors (C5) may lose some of the motor units but still be able to produce partial strength using the remaining motor units, which will expand the motor unit size to increase strength production with time.[10] On the other hand, partial loss of function at the C8 level may drastically impair some distal hand muscles because each hand muscle is innervated by a small number of motor units, which strongly rely on the CST projections. Another evidence of redundancy in the innervation of proximal upper limb muscles is the above-mentioned contribution of several levels of the spinal cord to proximal upper limb muscles compared to distal hand muscles.[11] Thus, in the distal hand muscles, there is no opportunity for expansion of the motor unit size at the muscle level, nor redundancy or alternative pathways at the spinal cord level – given the reliance on direct CST projections. Together, this evidence may explain why it is so hard to regain strength and predict the strength recovery in distal hand muscles.

Additional limitations

The following factors were not considered in the analysis.

(1) other factors related to muscle anatomy and mechanics, such as pennation angle, fiber type composition, and cross-sectional area;

(2) control for the rehabilitation provided to each individual during the natural recovery process - it is known that rehabilitative efforts initially focus on improving the proximal upper limb strength, which initially involves training anti-gravity muscles as the individuals learn how to transfer during the activities of daily living. This is a limitation of the EMSCI dataset and should be addressed in future studies. Nonetheless, it has been shown in stroke that recovery of motor impairment in patients with intact CST is proportional to initial impairment and unaffected by upper-limb therapy dose – among other factors.[12] Specifically, our findings of MEP amplitude increase over time warrant further studies investigating if this phenomenon is related to the amount of rehabilitation delivered to each individual.

(3) account for other anatomical systematic differences between subgroups, such as spinal syndromes (*e.g.,* central cord or Brown-Sequard syndromes).

Materials and methods used in the electrophysiological multimodal assessments of the hand muscles.

Motor evoked potentials

TMS was used to quantify the CST and LMN integrity by the motor evoked potential (MEP) amplitude and latency measured using sEMG of the *abductor digiti minimi* muscle at both sides of the body. The following TMS stimulation parameters were used: (Coil) double cone if available, or else ring-shaped pancake coil; (Position) Double Cone was 45° inclined to the contralateral side over C3 or C4 respectively (M1 was identified by C3 and C4 of the 10/20 EEG system); (Position) Ring-shaped pancake coil placed over C3, inclined 30-45° to the contralateral side and placed over C4, 30-45° inclined to the contralateral side; (Background sEMG) isotonic contraction of the *abductor digiti minimi* muscle (20% of maximal contraction) where possible; (Intensity) threshold 1,5 fold. For recording the MEP at the *abductor digiti minimi* muscle: (Electrodes) surface electrodes; (Positioning) active electrodes were over muscle body of the *abductor digiti minimi* muscle and V metacarpophalangeal joint; (Ground electrode) between stimulation and recording; (Impedance) < 5kOhm; (Filters) bandpass between 10Hz-2000Hz; (Recording time) 100ms; (Reproductions) 3-5 clear reproductions (latency variations within 0.5 ms; amplitude variations within 20%). Data analysis included latency (stimulation-onset in ms of the fastest response) and amplitude (baseline to maximal negative peak in μV of the largest amplitude). The values ranged from 10-50ms (latency) and 0-20mV (Amplitude).

Somatosensory evoked potentials

EEG was used to quantify SSEP amplitude and latency after neuromuscular electrical stimulation (NMES) of the ulnar nerve at both sides of the body. The following stimulation parameters were used: (Pulse) square wave with a pulse width = 0.2ms (increased up to 0.5ms in cases of unsatisfying responses) and frequency = 3Hz; (Intensity) motor threshold; (Stimulation site) ulnar nerve at the ulnar side of the pulse. For recording: (Electrodes) disposable needle electrodes; (Positioning): C3 (for stimulation on right pulse) or C4 (for stimulation on the left pulse) against Fz; (Ground) in between stimulation and recording; (Impedance) < 5kOhm; (Filters) Cortical = bandpass between 10Hz-2000Hz and Peripheral = bandpass between 50Hz-2000Hz; (Recording time) 100ms; (Reproducibility) > 1 clear reproduction (difference between the two sets of averaged responses no bigger than 0.5ms for latency and 20% for amplitudes). Data analysis included latency (minimal N20, corresponding P30 and N9, all in ms) and amplitude (N20/P30 in μV). The latency values ranged from 15-50ms (N20), 20-50ms (P25) and 52-25ms (N9); and the amplitude values between 0-10μV.

Nerve conduction studies

NMES was also used to quantify compound muscle action potential (CMAP) amplitude, nerve conduction velocity (NCV), and F-wave persistence in nerve conduction studies (NCS) at both sides of the body. Similar to SSEP, the ulnar nerve was electrically stimulated. The following stimulation parameters were used: (Stimulation) pulse with = 0.1-0.2ms (increase to 0.5ms in case of unsatisfying response); (Intensity) supra maximal; (Stimulation site) ventral wrist, ulnar sides [Cathode distal] and proximal sulcus ulnaris [Cathode distal]. For recording: (Electrodes) surface electrodes; (Positioning) active electrodes were over muscle body of the *abductor digiti minimi* muscle and the V metacarpophalangeal joint; (Ground) between stimulation and recording; (Impedance) < 5kOhm; (Filters) band-pass between 5-10.000Hz. Data analysis included latency

(distal motor latency: stimulation onset in ms), amplitude (CMAP amplitude: baseline to maximal negative Peak in mV) and NCV. The distal motor latency values ranged from 1.5-10ms, the CMAP amplitude from 0-50mV and the NCV from 20-100m/s. For the F-wave persistence analysis the same parameters were used except the filter (band-pass filter between 100-10.000Hz), 10-20 stimulations were conducted, and values ranged from 20-100ms (latency) and 0-100% (F-wave persistence). The values for F-wave persistence were a percentage of the total stimulations in which an F-wave could be elicited.

References

1. Kirshblum SC, Waring W, Biering-Sorensen F, et al. Reference for the 2011 revision of the International Standards for Neurological Classification of Spinal Cord Injury. *J Spinal Cord Med*. 2011;34(6):547-554. doi:10.1179/107902611X13186000420242
2. Kuypers HGJM. A New Look at the Organization of the Motor System. *Prog Brain Res*. 1982;57(C):381-403. doi:10.1016/S0079-6123(08)64138-2
3. Morecraft RJ, Ge J, Stilwell-Morecraft KS, McNeal DW, Pizzimenti MA, Darling WG. Terminal distribution of the corticospinal projection from the hand/arm region of the primary motor cortex to the cervical enlargement in rhesus monkey. *J Comp Neurol*. 2013;521(18):4205-4235. doi:10.1002/cne.23410
4. Morecraft RJ, Ge J, Stilwell-Morecraft KS, Rotella DL, Pizzimenti MA, Darling WG. Terminal organization of the corticospinal projection from the lateral premotor cortex to the cervical enlargement (C5–T1) in rhesus monkey. *J Comp Neurol*. 2019;527(16):2761-2789. doi:10.1002/cne.24706
5. Baker SN, Perez MA. Reticulospinal Contributions to Gross Hand Function after Human Spinal Cord Injury. *J Neurosci*. 2017;37(40 PG-9778-9784):9778-9784. doi:https://dx.doi.org/10.1523/JNEUROSCI.3368-16.2017
6. Jenny AB, Inukai J. Principles of motor organization of the monkey cervical spinal cord. *J Neurosci*. 1983;3(3):567-575. doi:10.1523/jneurosci.03-03-00567.1983
7. Phillips CG, Porter R. The Pyramidal Projection to Motoneurones of Some Muscle Groups of the Baboon's Forelimb. In: ; 1964:222-245. doi:10.1016/S0079-6123(08)60625-1
8. Palmer E, Ashby P. Corticospinal projections to upper limb motoneurones in humans. *J Physiol*. 1992;(448):397-412.
9. Gesslbauer B, Hruby LA, Roche AD, Farina D, Blumer R, Aszmann OC. Axonal components of nerves innervating the human arm. *Ann Neurol*. 2017;82(3):396-408. doi:10.1002/ana.25018
10. Li X, Jahanmiri-Nezhad F, Rymer WZ, Zhou P. An Examination of the Motor Unit Number Index (MUNIX) in muscles paralyzed by spinal cord injury. *IEEE Trans Inf Technol Biomed*. 2012;16(6 PG-1143-9):1143-1149. doi:https://dx.doi.org/10.1109/TITB.2012.2193410
11. Schirmer CM, Shils JL, Arle JE, et al. Heuristic map of myotomal innervation in humans using direct intraoperative nerve root stimulation: Clinical article. *J Neurosurg Spine*. 2011;15(1):64-70. doi:10.3171/2011.2.SPINE1068
12. Stinear CM, Byblow WD, Ackerley SJ, Smith MC, Borges VM, Barber PA. Proportional Motor Recovery after Stroke: Implications for Trial Design. *Stroke*. 2017;48(3):795-798. doi:10.1161/STROKEAHA.116.016020

Supplementary Figures and Tables

Supplementary Figure 1

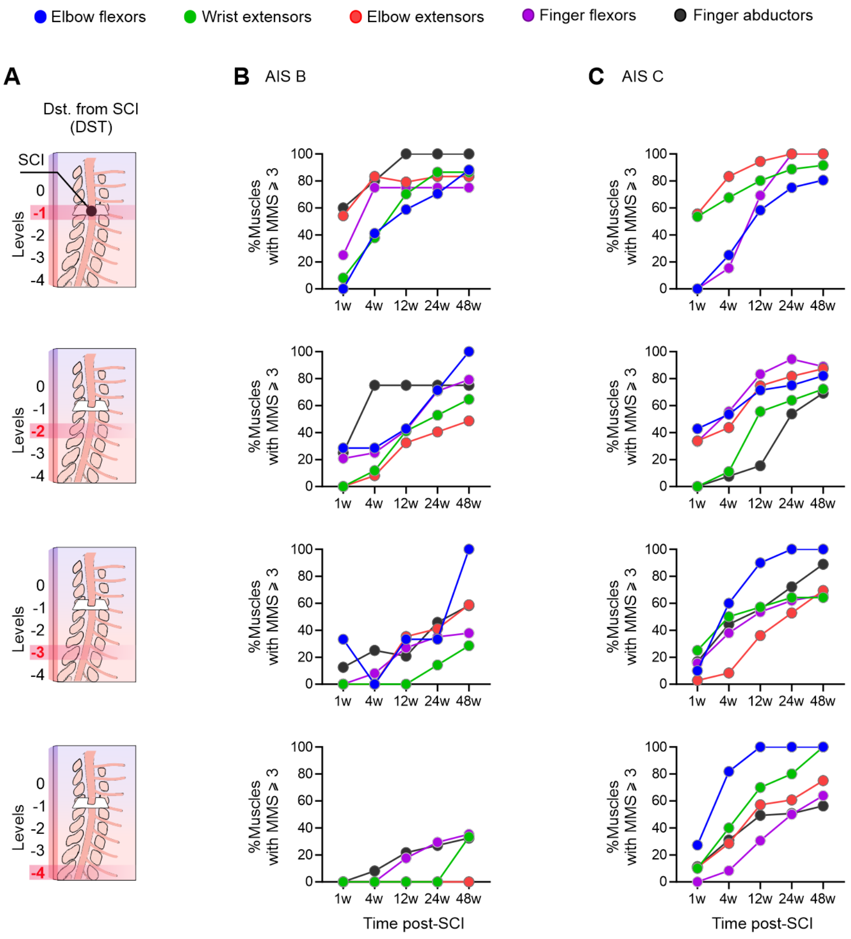

Supplementary Figure 1. Strength recovery in upper limb muscles after cervical SCI. (**A**) The distance between the SCI and upper limb muscles (DST) was controlled in panels B, C. (**B, C**) In individuals classified as AIS B or C, the probability of the proximal muscles (*i.e.* elbow flexors, wrist extensors, elbow extensors) achieve against gravity strength (MS ≥ 3) was greater compared to distal muscles (*i.e.*, finger flexors and abductors) – especially if the respective muscle is distant from the SCI (*i.e.*, levels -3 and -4).

Supplementary Figure 2

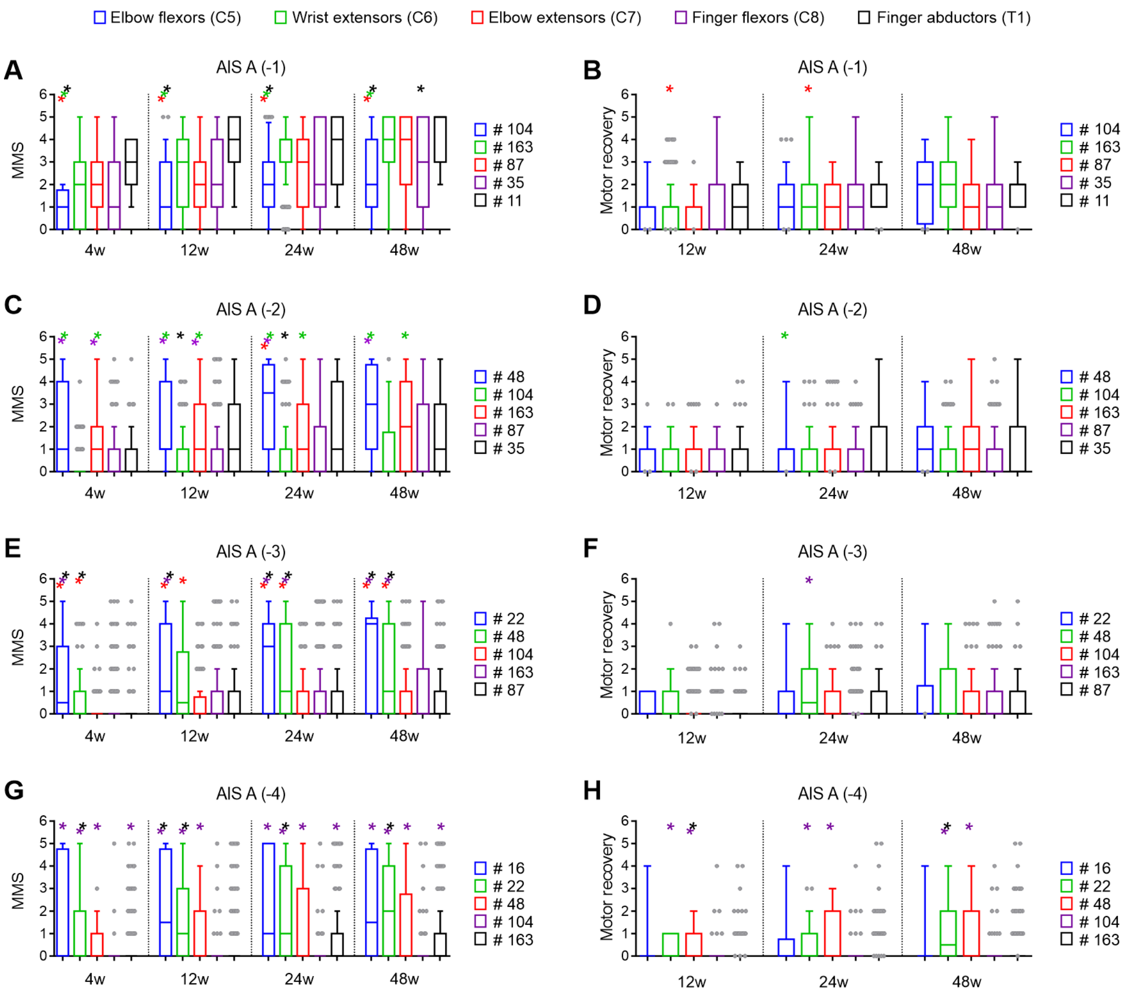

Supplementary Figure 2. Motor recovery of muscles below the motor level of injury in AIS A individuals. (**A**) Muscles that are one level below the injury (-1) show distinct responses after the injury, the elbow flexors were more impaired. (**B**) All muscles at this distance from the injury recover to a similar extent 48 weeks post-SCI. (**C**) When muscles are two levels below the injury (-2) the SCI seems to display more effect on distal muscles such as finger flexors and abductors. (**D**) All muscles at this distance from the injury recover to a similar extent 48 weeks post-SCI. (**E, G**) The effect described in **D** becomes more apparent when the muscles are further below the level of injury (-3 or -4 levels), the lesion has more impact over distal hand muscles in comparison to proximal muscles. (**F, H**) Strength recovery of these muscles seems to occur more at wrist and elbow extensors compared to finger flexors and abductors (**H**). Kruskal-Wallis test with Dunn's multiple comparisons test: *$P < 0.05$ relative to elbow flexors (*blue), wrist extensors (*green), elbow extensors (*red), finger flexors (*purple) or finger abductors (*black). Note: data are median (line in the middle of the box), the first quartile forms the bottom, and the third quartile forms the top of the box, whiskers represent the 5-95 percentiles; any data beyond the whiskers are shown as points (grey dots); the numbers on the right of each panel indicates the number of muscles available for each analysis. SCI = Spinal Cord Injury; AIS = American Spinal Cord Injury Association Impairment Scale; MMS = Muscle Motor Score; C = Cervical level; T = Thoracic level.

Supplementary Figure 3

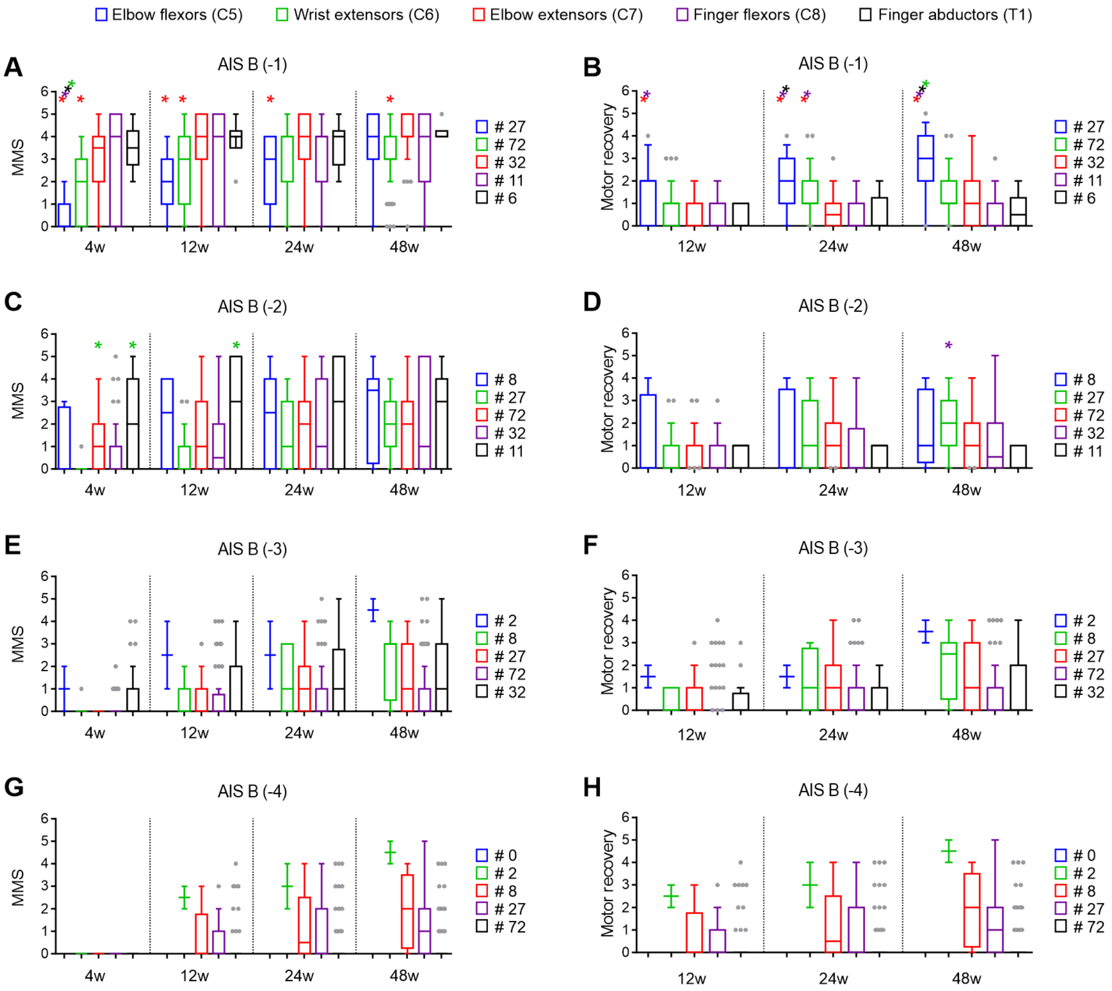

Supplementary Figure 3. Motor recovery of muscles below the motor level of injury in AIS B individuals. (**A**) Muscles that are one level below the injury (-1) show distinct responses after the injury, the elbow flexors are more impaired. (**B**) In AIS B individuals the proximal muscles that are one level below the injury recover the most, especially the elbow flexors. (**C**) When muscles are two levels below the injury (-2) a similar trend emerges where the proximal elbow flexors and the distal wrist extensors are more affected, but also (**D**) recover to some extent. (**E-H**) No statistical analysis was conducted for muscles 3 or 4 levels (-3 or -4) below the injury because of the reduced sample size for some muscles. Nonetheless, the trends indicate a similar behavior to AIS A individuals, where distal muscles such as the finger flexors and abductors are more impaired concerning more proximal muscles. Kruskal-Wallis test with Dunn's multiple comparisons test: *P < 0.05 relative to elbow flexors (*blue), wrist extensors (*green), elbow extensors (*red), finger flexors (*purple) or finger abductors (*black). Note: data are median (line in the middle of the box), the first quartile forms the bottom, and the third quartile forms the top of the box, whiskers represent the 5-95 percentiles; any data beyond the whiskers are shown as points (grey dots); the numbers on the right of each panel indicates the number of muscles available for each analysis. SCI = Spinal Cord Injury; AIS = American Spinal Cord Injury Association Impairment Scale; MMS = Muscle Motor Score; C = Cervical level; T = Thoracic level.

Supplementary Figure 4

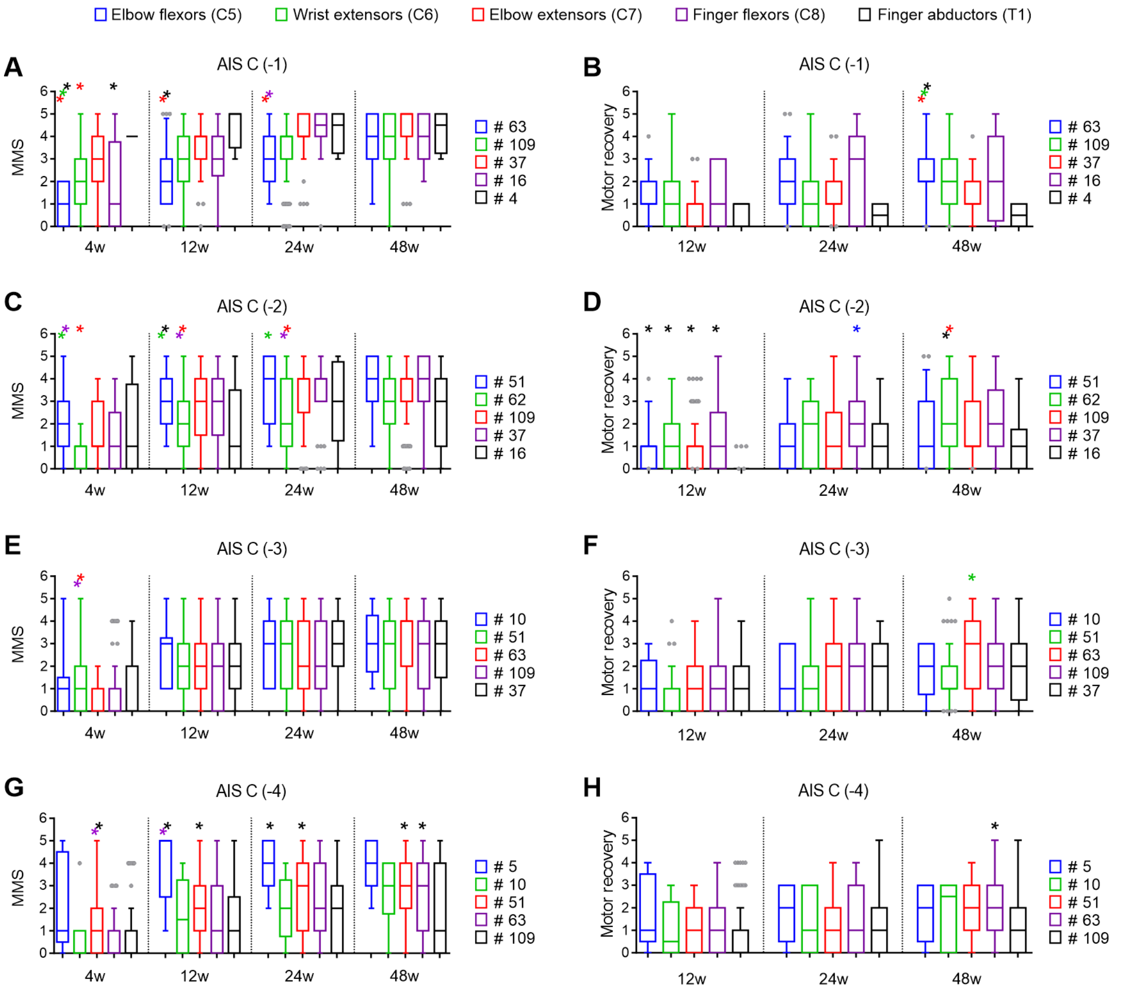

Supplementary Figure 4. Motor recovery of muscles below the motor level of injury in AIS C individuals. (**A**) Muscles that are one level below the injury (-1) show distinct responses after the injury, the elbow flexors were more impaired and (**B**) recover to a greater extent compared to wrist extensors and finger abductors. (**C**) When muscles are 2 levels below the injury (-2) elbow flexors are less affected early after the injury, but all muscles recover to a similar grade 48 weeks post-SCI. (**D**) Early after the injury, all muscles recover more than the finger abductors. The wrist extensors show a greater recovery compared to elbow extensors and finger abductors 48 weeks post-SCI. (**E**) When muscles are 3 levels below the injury (-3) the wrist extensors are less affected early after the injury, but all muscles recover to a similar grade 48 weeks post-SCI. (**F**) Muscle strength recovery is greater for elbow extensors compared to wrist extensors. (**G**) When the lesion is distant from the muscles level (*i.e.*, -4 levels) the proximal elbow flexors and extensors are less affected compared to finger flexors and abductors. (**H**) Muscle strength recovery is greater for the finger flexors compared to finger abductors 48 weeks post-SCI. Kruskal-Wallis test with Dunn's multiple comparisons test: **P* < 0.05 relative to elbow flexors (*blue), wrist extensors (*green), elbow extensors (*red), finger flexors (*purple) or finger abductors (*black). Note: data are median (line in the middle of the box), the first quartile forms the bottom, and the third quartile forms the top of the box, whiskers represent the 5-95 percentiles; any data beyond the whiskers are shown as points (grey dots); the numbers on the right of each panel indicates the number of muscles available for each analysis. SCI = Spinal Cord Injury; AIS = American Spinal Cord Injury Association Impairment Scale; MMS = Muscle Motor Score; C = Cervical level; T = Thoracic level.

Supplementary Figure 5

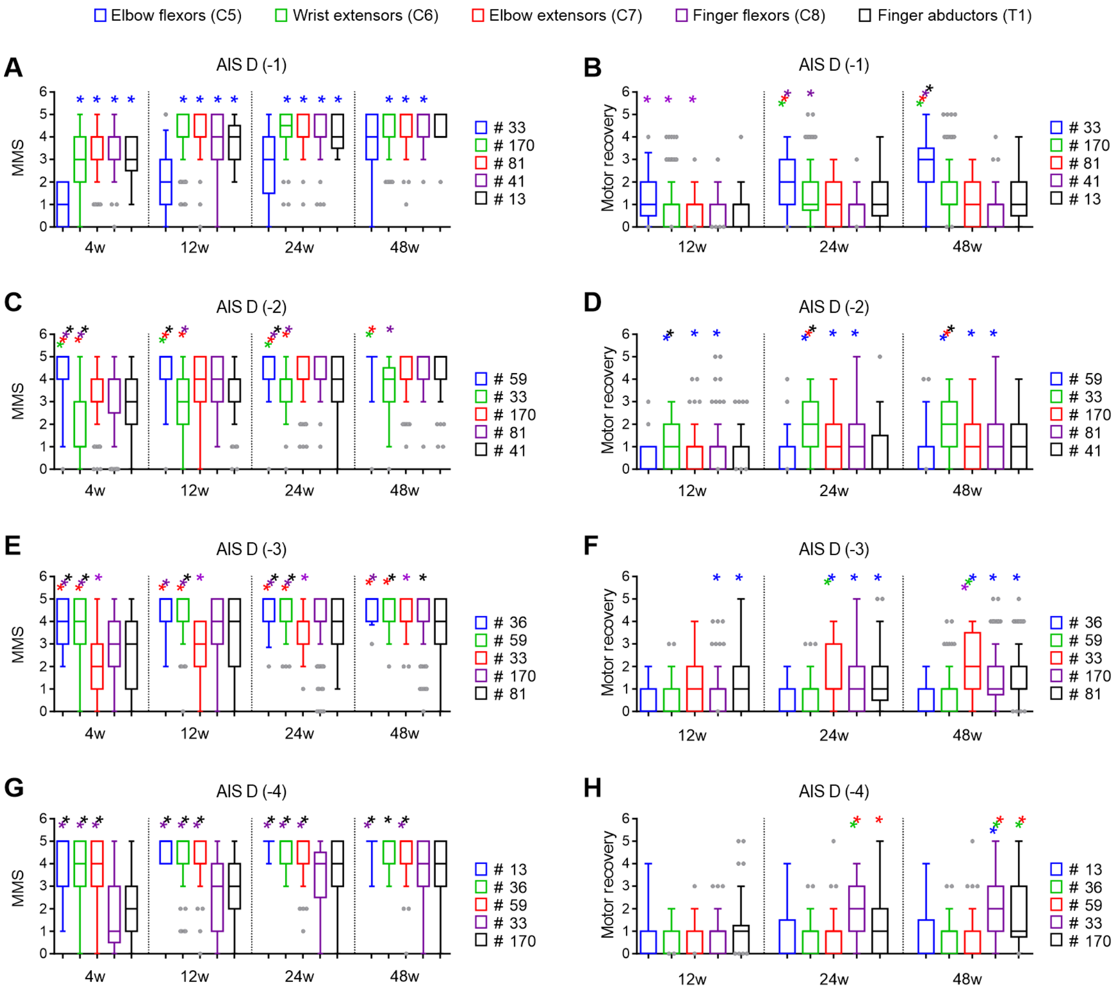

Supplementary Figure 5. Motor recovery of muscles below the motor level of injury in AIS D individuals. (**A**) Muscles that are one level below the injury (-1) show distinct responses after the injury, the elbow flexors are more impaired compared to all other muscles. (**B**) The elbow flexors also recover the most, compared to all other muscles 48 weeks post-SCI. (**C**) If the lesion is 2 levels above the muscle level (-2), elbow flexors are less affected and wrist extensors are more affected but also (**D**) recover to a greater extent compared to elbow extensors and finger abductors (note the differences in motor score recovery concerning elbow flexors should be interpreted with caution because of ceiling effects). (**E, F**) When the SCI is 3 or 4 levels above the muscle level (-3 or -4), the distal muscles such as the finger flexors and abductors are more impaired concerning the proximal muscles. (**G, H**) These distal muscles also recover to a greater extent compared to proximal muscles (note the differences in motor score recovery concerning elbow flexors should be interpreted with caution because of ceiling effects). Kruskal-Wallis test with Dunn's multiple comparisons test: **P* < 0.05 relative to elbow flexors (*blue), wrist extensors (*green), elbow extensors (*red), finger flexors (*purple) or finger abductors (*black). Note: data are median (line in the middle of the box), the first quartile forms the bottom, and the third quartile forms the top of the box, whiskers represent the 5-95 percentiles; any data beyond the whiskers are shown as points (grey dots); the numbers on the right of each panel indicates the number of muscles available for each analysis. SCI = Spinal Cord Injury; AIS = American Spinal Cord Injury Association Impairment Scale; MMS = Muscle Motor Score; C = Cervical level; T = Thoracic level.

Supplementary Figure 6

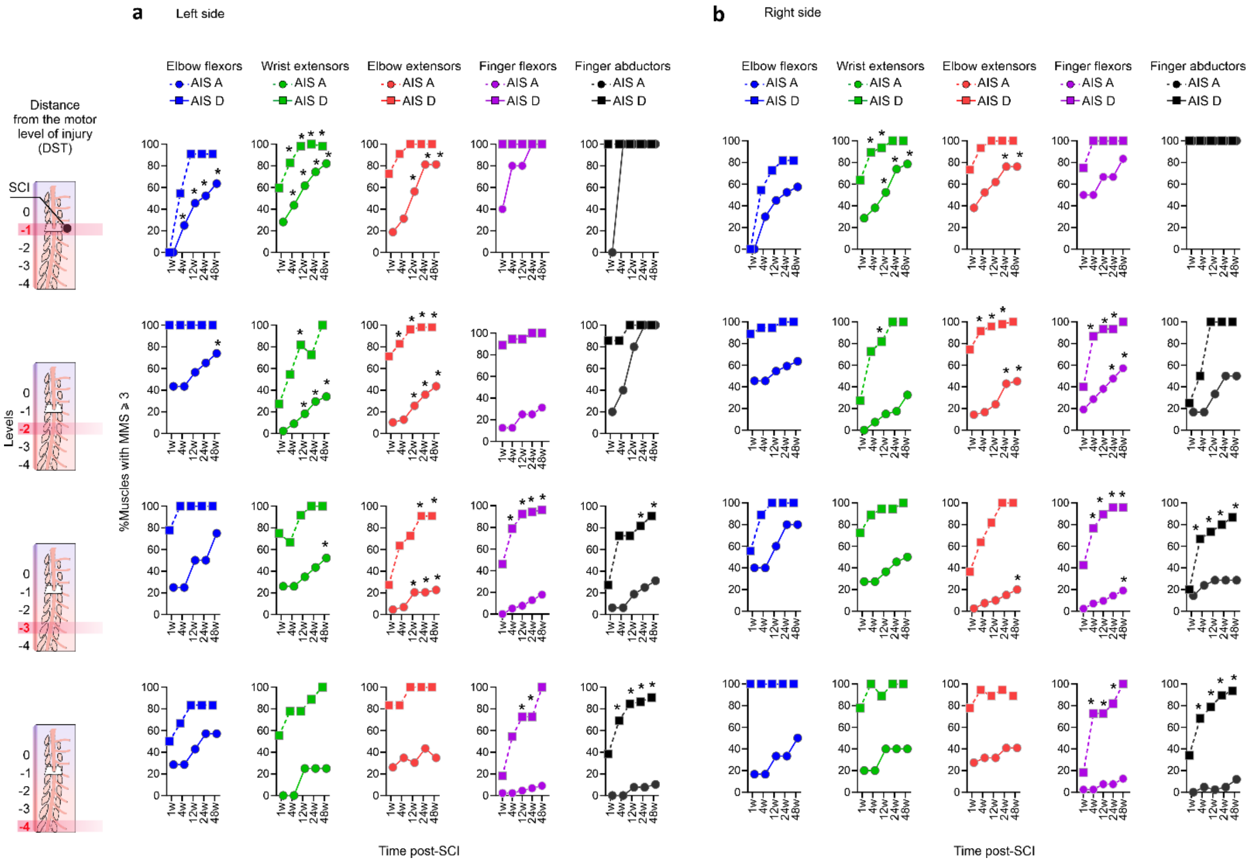

Supplementary Figure 6. Strength recovery in the left (a) and right (b) upper limb muscles after cervical SCI. The distance between the motor level and the myotome (DST) was controlled in panels a,b. **a,b** Similar results were evident for the left and right upper limb muscles. In individuals classified as AIS A, the probability of the proximal muscles (*i.e.* elbow flexors, wrist extensors, and elbow extensors) achieving against gravity strength (MMS ≥ 3) was greater compared to hand muscles (*i.e.*, finger flexors and abductors) – especially if the hand muscles are distant from the SCI (*i.e.*, levels -3 and -4). Hand muscles also took longer to regain strength in individuals classified as AIS A.

Notes: Data is % of muscles with an MMS ≥ 3. N = 476 (AIS A) and 362 (AIS D) muscles in **a**; N = 486 (AIS A) and 367 (AIS D) muscles in **b**; N = number of biologically independent samples; *P < 0.05, McNemar's tests (two-sided). SCI = Spinal Cord Injury; AIS = American Spinal Cord Injury Association Impairment Scale; MMS = Muscle Motor Score.

Supplementary Figure 7

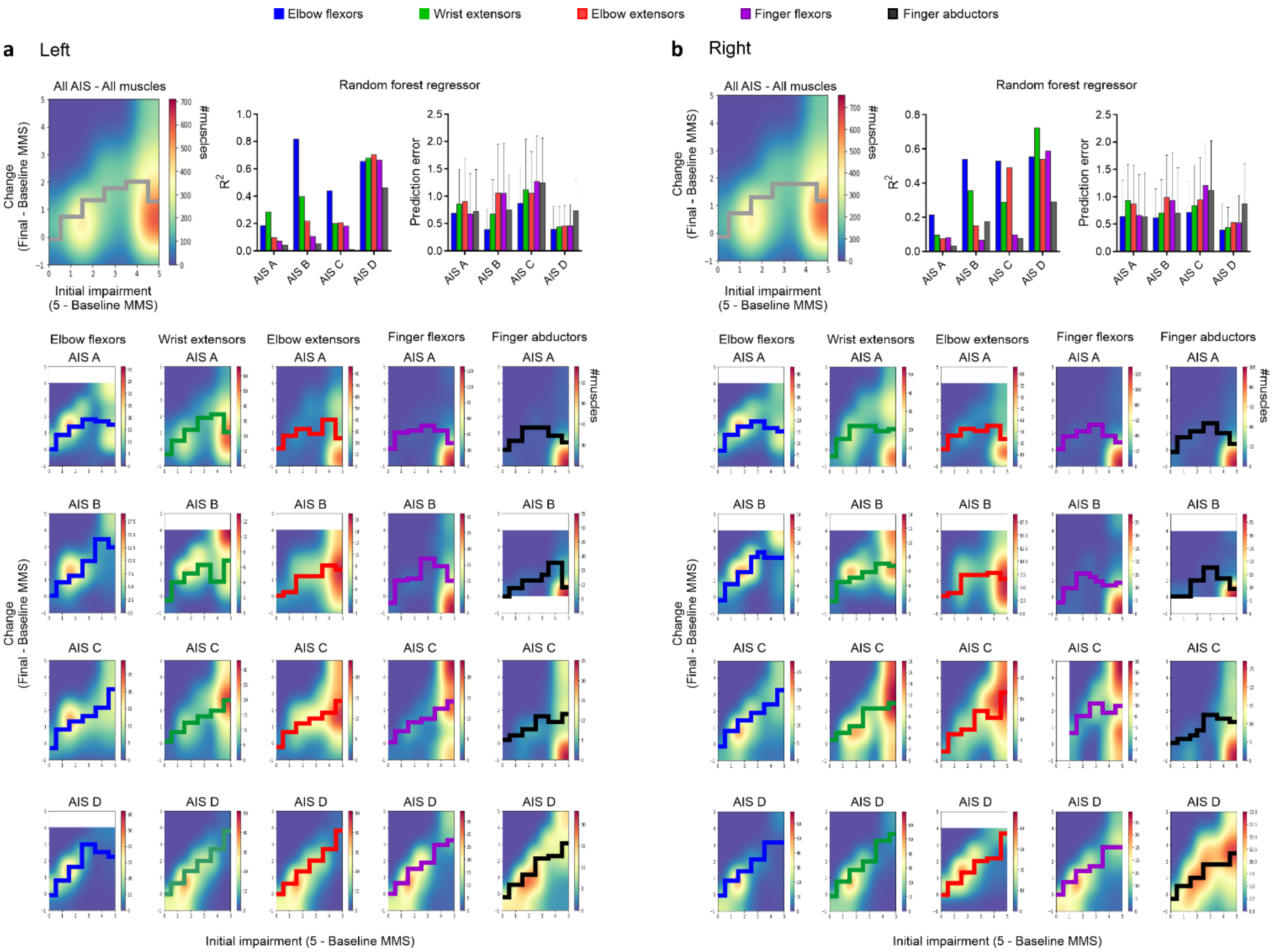

Supplementary Figure 7. Prediction of strength recovery after cervical SCI for the upper limb muscles on the left (a) and right (b) sides of the body. Similar results were evident for the left and right upper limb muscles. Baseline MMS is a good predictor of strength recovery at 1-year post-SCI for individuals with AIS D (high R^2 values) but is in most cases a poor predictor for those with an AIS A/B/C. Summary of the non-linear regression using random forest regressors indicates good prediction of strength recovery for all muscles of AIS D participants with a prediction error of ≈ 0.5 points. Although the prediction is fair to good for some of the proximal muscles in individuals with an AIS A/B/C, predicting late strength recovery solely based on the initial motor impairment is poor for distal hand muscles (R^2 ≈ 0.1).

Notes: Complex analysis using random forest regressor with 50% of the dataset for training and 50% for testing with 100 trees (estimators) in **a,b**. Data is Mean or Mean ± SD in upper panels (bar graphs). N = 2738 muscles in **a** and 2766 muscles in **b**. N = number of biologically independent samples; SCI = Spinal Cord Injury; AIS = American Spinal Cord Injury Association Impairment Scale; MMS = Muscle Motor Score.

Supplementary Figure 8

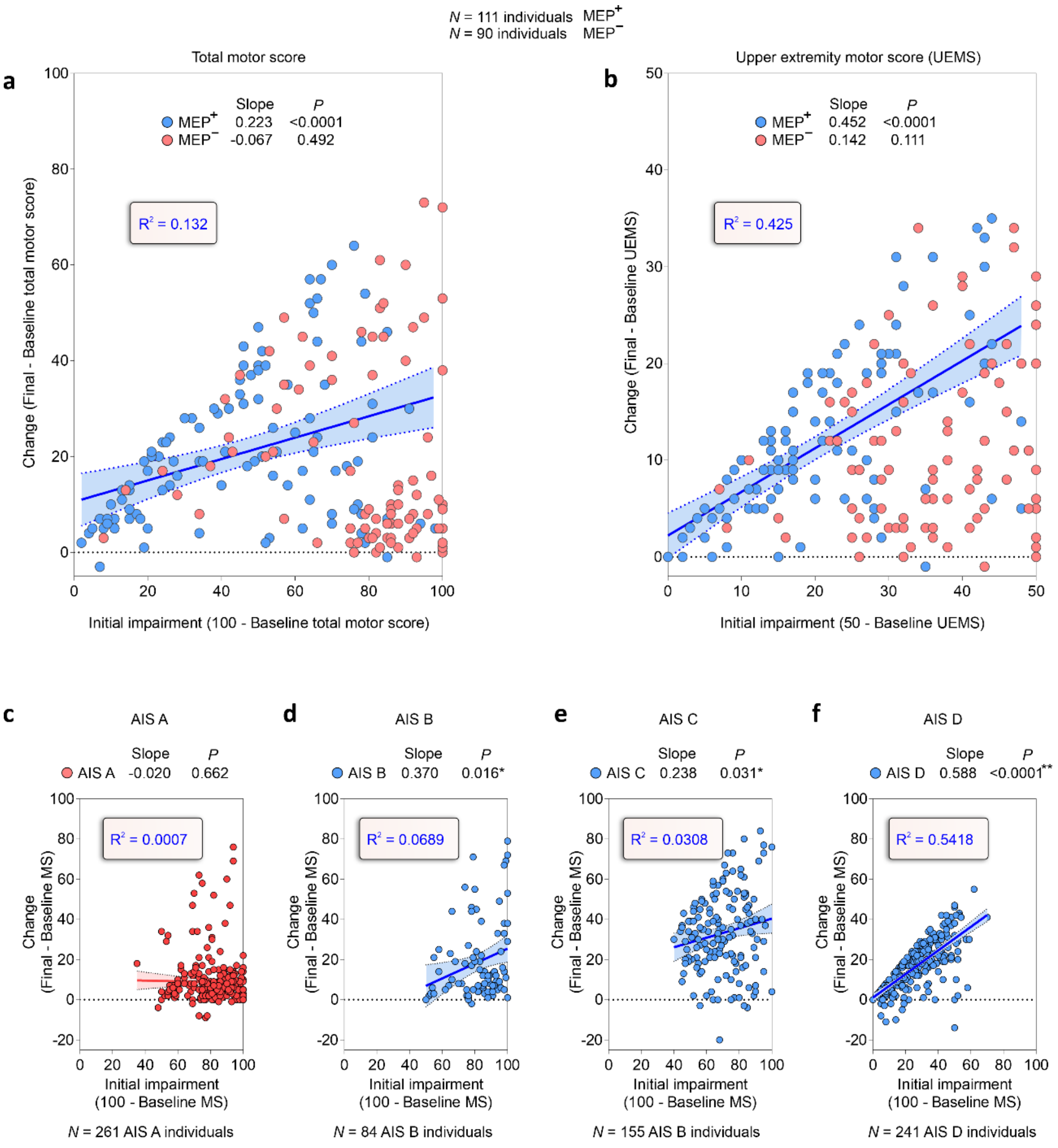

Supplementary Figure 8. CST and LMN integrity (as assessed by MEP) indicates impairment and recovery of the upper extremity after SCI. a Individuals with absent MEP (MEP^-) display greater damage to the descending pathway (evidenced by the greater initial impairment) and limited recovery of motor function of the spinal cord (*P* = 0.492). The presence of an MEP (MEP^+) indicates variable levels of spinal cord or LMN damage and recovery (*P* < 0.0001). **b** This effect is more apparent when considering the impairment and recovery of the upper extremity (upper extremity motor score, UEMS). The presence of an MEP (MEP^+) indicates proportional recovery of the UEMS (*P* < 0.0001), with better goodness of fit compared to the model described in panel **a**. **c-f** Recovery versus initial impairment in AIS A-D.

Notes: *P < 0.05 simple linear regression (slope is significantly non-zero). The shaded area represents the 95% confidence interval of the regression line. Two individuals were excluded from the total MEP sample (203) because

of absent AIS classification at baseline. Note: SCI = Spinal Cord Injury; UEMS = Upper Extremity Motor Score; AIS = American Spinal Cord Injury Association Impairment Scale; MEP = Motor Evoked Potential.

Supplementary Table 1

Supplementary Table 1. Demographics and baseline clinical parameters.

**Demographics**		**Full sample**	**Very acute**	**MEP sample**	**SSEP sample**	**NCS sample**
n		748	440	203	313	280
Gender		599 M, 149 F	343 M, 97 F	171 M, 32 F	254 M, 59 F	228 M, 52 F
Age (Mean ± SD)		46.5 ± 18.9	47.1 ± 19.1	43.8 ± 18.8	44.4 ± 18.8	45.0 ± 19.0
**Baseline Clinical parameters**		**Full sample**	**Very acute**	**MEP sample**	**SSEP sample**	**NCS sample**
Neurological level of injury	C1	22	24	4	7	7
	C2	45	23	4	8	9
	C3	95	62	28	40	38
	C4	279	168	81	120	109
	C5	189	88	46	83	70
	C6	72	33	25	38	31
	C7	33	7	14	15	14
	C8	13	4	1	2	2
	NT	0	31	0	0	0
AIS grade	A	261	153	56	87	77
	B	84	51	31	52	45
	C	155	98	44	66	54
	D	241	121	71	107	103
	NT	7	17	1	1	1

MEP = motor evoked potential; SSEP = somatosensory evoked potential; NCS = nerve conduction studies; M = male; F = female; SD = standard deviation; C = cervical level; AIS = american spinal injury association impairment scale; NT = not tested.

Supplementary Table 2

Supplementary Table 2. Scoring of electrophysiological examinations (Hupp, *et al.*, 2018).

Modality	Parameter	Scoring 2	Scoring 1	Scoring 0
MEP	Latency	Not scored	< 25 ms	≥ 25 ms or abolished
	Amplitude	≥ 0.1 mV	< 0.1 mV	Abolished
SSEP	Latency	Not scored	< 21.7 ms	≥ 21.7 ms or abolished
	Amplitude	≥ 0.5 uV	< 0.5 uV	Abolished
NCS	Amplitude	≥ 0.4 mV	< 0.4 mV	Abolished
	F-wave persistance	Not scored	> 50 %	≤ 50 %

Note: Electrophysiological examinations were scored according to the latency and amplitude in motor evoked potentials (MEP) and somatosensory evoked potentials (SSEP), respectively. Nerve conduction studies (NCS) were scored according to amplitude of compound muscle action potentials (CAMP) and F-wave persistence.

Supplementary Table 3

Supplementary Table 3. Feature importance in the supervised machine learning models.

**All AIS and muscles (Figure 5B)**	**(without muscle)**		**(with muscle)**
AIS	0.24707	AIS	0.20526
MMS	0.25608	Muscle	0.1721
DST	0.19587	MMS	0.20074
LT	0.18199	DST	0.15662
PP	0.11898	LT	0.16093
		PP	0.10435
**AIS A (Figure 5C, D)**	**(without muscle)**		**(with muscle)**
MMS	0.41186	Muscle	0.21887
DST	0.31671	MMS	0.31242
LT	0.12158	DST	0.23116
PP	0.14986	LT	0.11251
		PP	0.12504
**AIS B (Figure 5C, D)**	**(without muscle)**		**(with muscle)**
MMS	0.29859	Muscle	0.3172
DST	0.37651	MMS	0.20715
LT	0.15386	DST	0.20443
PP	0.17104	LT	0.14191
		PP	0.1293
**AIS C (Figure 5C, D)**	**(without muscle)**		**(with muscle)**
MMS	0.29486	Muscle	0.23575
DST	0.32577	MMS	0.24145
LT	0.18009	DST	0.22097
PP	0.19928	LT	0.14633
		PP	0.1555
**AIS D (Figure 5C, D)**	**(without muscle)**		**(with muscle)**
MMS	0.35449	Muscle	0.25555
DST	0.35876	MMS	0.23421
LT	0.12353	DST	0.28099
PP	0.16322	LT	0.09585
		PP	0.1334

Note: Feature importance was calculated using 50% of the dataset for training and 50% for testing. AIS = American Spinal Cord Injury Association Impairment Scale; MMS = Muscle Motor Score; DST = Distance from the motor level of injury; LT = Light Touch sensation; PP = Pin Prick sensation.

Supplementary Table 4

Supplementary Table 4. Leave-one-subject-out cross-validation.

	AUC (PR)	AUC (ROC)
Model 1 (Figure 5b)	0.692	0.876
Model 1+ (Figure 5b)	0.678	0.868
Model 1 - AIS A (Figure 5c)	0.802	0.734
Model 1 - AIS B (Figure 5c)	0.840	0.677
Model 1 - AIS C (Figure 5c)	0.896	0.510
Model 1 - AIS D (Figure 5c)	0.908	0.502
Model 1+ - AIS A (Figure 5d)	0.791	0.720
Model 1+ - AIS B (Figure 5d)	0.845	0.686
Model 1+ - AIS C (Figure 5d)	0.888	0.504
Model 1+ - AIS D (Figure 5d)	0.907	0.517
Model 2 (Figure 5e)	0.921	0.578
Model 3 (Figure 5e)	0.893	0.592
Model 4 (Figure 5e)	0.859	0.646
Model 5 (Figure 5e)	0.827	0.676
Model 6 (Figure 5e)	0.756	0.640

Note: AUC = Area Under the Curve in adimensional units; PR = Precision-Recall; ROC = Receaver Operator Characteristics.

Supplementary Table 5

Supplementary Table 5. Leave-one-subject-out cross-validation.

	AUC (PR)	AUC (ROC)	Accuracy	p value (single-tailed)
**MEP**				
Model 6 (Figure 6c)	0.672	0.625	0.640	**p = 0.049***
Model 7 (Figure 6c)	0.738	0.694	0.702	
**SSEP**				
Model 6 (Figure 6d)	0.645	0.620	0.649	p = 0.140
Model 8 (Figure 6d)	0.688	0.662	0.681	
**NCS**				
Model 6 (Figure 6e)	0.681	0.655	0.694	**p = 0.009***
Model 9 (Figure 6e)	0.606	0.590	0.612	

*Note: MEP = Motor Evoked Potential; SSEP = Somatosensory Evoked Potential; NCS = Nerve Conduction Studies; AUC = Area Under the Curve in adimensional units; PR = Precision-Recall; ROC = Receaver Operator Characteristics. *$p < 0.05$ (single-tailed) McNemar test.*
