# Supplementary material for: Segmental motor recovery after cervical spinal cord injury relates to density and integrity of corticospinal tract projections
Source: Nat Commun. 2023 Feb 9;14:723. doi: 10.1038/s41467-023-36390-7 (PMC9911610; doi:10.1038/s41467-023-36390-7)
Supplement: Supplementary file 3 — Description of Additional Supplementary Files [file 41467_2023_36390_MOESM3_ESM.pdf]

Description of Additional Supplementary Files

Supplementary Software 1. Codes utilized for pre-processing, *i.e.*, the transformation of wide to long data (Code 1 in LabVIEW®), and machine learning analysis using random forest regressors (Figure 4) and random forest classifiers with leave one muscle out cross-validation (Figures 5 and 6) (Code 2 in Python).
