# Supplementary material for: Segmental motor recovery after cervical spinal cord injury relates to density and integrity of corticospinal tract projections
Source: Nat Commun. 2023 Feb 9;14:723. doi: 10.1038/s41467-023-36390-7 (PMC9911610; doi:10.1038/s41467-023-36390-7)
Supplement: Supplementary file 4 — Supplementary Software 1 [file 41467_2023_36390_MOESM4_ESM.zip › Gustavo-Balbinot-Segmental-analysis-in-cervical-SCI-reveals-the-recovery-potential-of-hand-muscles-with-preserved-CST-f7d60e4/Code 2 - Data analysis - Machine learning regressors and classifiers (LOOCV).docx]

**Data analysis (Python): Randon forest regressors (Figure 4)**

# -*- coding: utf-8 -*-

"""

Created on Wed Mar 3 08:00:24 2021

@author: Gush

"""

import pandas as pd

import numpy as np

import matplotlib.pyplot as plt

import statistics

dataset = pd.read_csv(r'C:\Users\gusta\Desktop\Quarantene work 28.05.21\ASIA conference - 2022 - New Orleans\Abstract 1 - Recovery profiles FEST\Recovery profile X DST for heatmap.txt', delimiter ='\t')

X = dataset.iloc[:, 0].values

y = dataset.iloc[:, 1:3].values

print(y)

from sklearn.model_selection import train_test_split

X_train, X_test, y_train, y_test = train_test_split(X, y, test_size=0.5, random_state=0)

from sklearn.ensemble import RandomForestRegressor

regressor = RandomForestRegressor(n_estimators=100, random_state=0)

regressor.fit(X, y)

y_pred = regressor.predict(X_test)

plt.scatter(X, y, color = 'blue')

X_grid = np.arange(min(X), max(X), 0.01)

X_grid = X_grid.reshape((len(X_grid), 1))

plt.plot(X_grid, regressor.predict(X_grid),

color = 'red')

plt.title('Random Forest Regression')

plt.xlabel('Initial impairment (5 - baseline MS)')

plt.ylabel('Change (final - baseline MS)')

plt.show()

from sklearn.metrics import r2_score

R2 = r2_score(y_test, y_pred)

Pred_error = y_test-y_pred

Abs_Pred_error = abs(Pred_error)

Average_Prediction_error = sum(Abs_Pred_error)/len(Abs_Pred_error)

SD_Average_Prediction_error = statistics.stdev(Abs_Pred_error)

print (Abs_Pred_error)

print ('R2 score =', R2)

print('Average prediction error =', Average_Prediction_error)

print ('Error SD =', SD_Average_Prediction_error)

**Data analysis (Python): Leave one muscle out cross-validation code (Figures 5 and 6)**

# -*- coding: utf-8 -*-

"""

Created on Thu Jan 21 14:05:36 2021

@author: Gush

"""

import pandas as pd

import numpy as np

dataset = pd.read_csv(r'C: path', delimiter ='\t')

print(dataset)

X = dataset.iloc[:, 0:4].values

print(X)

y = dataset.iloc[:, 4].values

from sklearn.model_selection import train_test_split

X_train, X_test, y_train, y_test = train_test_split(X, y, test_size=0.5, random_state=0)

from sklearn.ensemble import RandomForestClassifier

regressor = RandomForestClassifier(n_estimators=100, random_state=0)

regressor.fit(X_train, y_train)

y_pred = regressor.predict(X_test)

from sklearn.metrics import classification_report, confusion_matrix, accuracy_score

print(confusion_matrix(y_train,y_pred))

print(classification_report(y_train,y_pred))

print(accuracy_score(y_train, y_pred))

print(y_test)

print(y_pred)

target_names = ['No rec', 'Rec'];

#output_dict make the report saved as a dictionary

A=classification_report(y_test, y_pred, target_names=target_names, output_dict=True)

B=confusion_matrix(y_test, y_pred)

#Read values as

class_0_f1score=A['No rec']['f1-score']

print(A)

print(B)

#Leave one out cross validation

from sklearn.model_selection import LeaveOneOut

X = dataset.iloc[:, 0:4].values

y = dataset.iloc[:, 4].values

loo = LeaveOneOut()

loo.get_n_splits(X)

print(loo)

LeaveOneOut()

LOOCV_pred = []

for train_index, test_index in loo.split(X):

print("TRAIN:", train_index, "TEST:", test_index)

X_LOOCV_train, X_LOOCV_test = X[train_index], X[test_index]

y_LOOCV_train, y_LOOCV_test = y[train_index], y[test_index]

print(X_LOOCV_train, X_LOOCV_test, y_LOOCV_train, y_LOOCV_test)

LOOCV_pred.append(y_LOOCV_test)

print('y_test =', y)

print('y_pred =', LOOCV_pred)

import csv

LOOCV_output = open(r'C: path', 'w+', newline ='')

write = csv.writer(LOOCV_output)

write.writerow('y_test')

write.writerow(y)

write.writerow('y_pred')

write.writerow(LOOCV_pred)
