# Supplementary material for: Segmental motor recovery after cervical spinal cord injury relates to density and integrity of corticospinal tract projections
Source: Nat Commun. 2023 Feb 9;14:723. doi: 10.1038/s41467-023-36390-7 (PMC9911610; doi:10.1038/s41467-023-36390-7)
Supplement: Supplementary file 5 — Reporting Summary [file 41467_2023_36390_MOESM5_ESM.pdf]

nature portfolio

Corresponding author(s): Gustavo Balbinot and Jose Zariffa

Last updated by author(s): Jan 16, 2023

Reporting Summary

Nature Portfolio wishes to improve the reproducibility of the work that we publish. This form provides structure for consistency and transparency in reporting. For further information on Nature Portfolio policies, see our Editorial Policies and the Editorial Policy Checklist.

Statistics

For all statistical analyses, confirm that the following items are present in the figure legend, table legend, main text, or Methods section.

n/a	Confirmed	
☐	☒	The exact sample size (*n*) for each experimental group/condition, given as a discrete number and unit of measurement
☐	☒	A statement on whether measurements were taken from distinct samples or whether the same sample was measured repeatedly
☐	☒	The statistical test(s) used AND whether they are one- or two-sided<br>*Only common tests should be described solely by name; describe more complex techniques in the Methods section.*
☐	☒	A description of all covariates tested
☐	☒	A description of any assumptions or corrections, such as tests of normality and adjustment for multiple comparisons
☐	☒	A full description of the statistical parameters including central tendency (e.g. means) or other basic estimates (e.g. regression coefficient) AND variation (e.g. standard deviation) or associated estimates of uncertainty (e.g. confidence intervals)
☐	☒	For null hypothesis testing, the test statistic (e.g. *F*, *t*, *r*) with confidence intervals, effect sizes, degrees of freedom and *P* value noted<br>*Give P values as exact values whenever suitable.*
☒	☐	For Bayesian analysis, information on the choice of priors and Markov chain Monte Carlo settings
☐	☒	For hierarchical and complex designs, identification of the appropriate level for tests and full reporting of outcomes
☒	☐	Estimates of effect sizes (e.g. Cohen's *d*, Pearson's *r*), indicating how they were calculated

Our web collection on statistics for biologists contains articles on many of the points above.

Software and code

Policy information about availability of computer code

Data collection	The data collection was performed according to clinical standards using commercially available software (see Neurophysiology Manual used within EMSCI, www.emsci.org).
Data analysis	The analysis was conducted using Python scikit-learn v 1.0.2 [machine learning analysis (random forest regressors and classifiers) and data visualization], SPSS® Statistics v.25 (descriptive analysis, median comparisons), Excel v. 16.68 (data sorting), LabVIEW® v. 2020 (data visualization and sorting), and GraphPad Prism® v.9.0.0 (121) (data visualization and descriptive analysis).<br><br>The custom source code utilized during data harmonization and complex analysis (machine learning) is deposited at https://github.com/Gustavo-Balbinot/Segmental-analysis-in-cervical-SCI-reveals-the-recovery-potential-of-hand-muscles-with-preserved-CST. Below is the code availability statement included in the manuscript.<br><br>Code availability<br>Source codes are provided with this paper (https://github.com/Gustavo-Balbinot/Segmental-analysis-in-cervical-SCI-reveals-the-recovery-potential-of-hand-muscles-with-preserved-CST).

For manuscripts utilizing custom algorithms or software that are central to the research but not yet described in published literature, software must be made available to editors and reviewers. We strongly encourage code deposition in a community repository (e.g. GitHub). See the Nature Portfolio guidelines for submitting code & software for further information.

Data

Policy information about availability of data

All manuscripts must include a data availability statement. This statement should provide the following information, where applicable:

- Accession codes, unique identifiers, or web links for publicly available datasets
- A description of any restrictions on data availability
- For clinical datasets or third party data, please ensure that the statement adheres to our policy

Deindentified participant source data utilized during the study are available upon publication of the study in a permanent public repository (https://www.synapse.org/#!Synapse:syn27778461/wiki/616964). This study conducted a secondary analysis of the European Multicenter Study about Spinal Cord Injury (EMSCI; NCT01571531) in accordance with the terms agreed upon the receipt of the dataset (REB #20-5914 – University Health Network). Additional related data is available upon reasonable request to EMSCI.

Human research participants

Policy information about studies involving human research participants and Sex and Gender in Research.

Reporting on sex and gender	We did not conduct post hoc sex- and gender-based analysis because the study design is insufficient (i.e., low prevalence of SCI among females) to enable meaningful conclusions (599 males and 149 females). Although the number of females is substantial, our analysis involves a series of stratifications that would reduce the ample size drastically. In addition, the pivotal MEP dataset contained only 32 females (171 males).
Population characteristics	The covariate-relevant characteristics were the ASIA classification, distance from the motor level of injury, strength and sensation, and electrophysiological multimodal assessments at baseline. Other co-variates such as (1) age (Mean 46.5 ± SD 18.9) and (2) gender (599 M, 149 F) are presented in Supplementary Table 1 but were not explored in the present study. (1) Age is not significantly correlated with motor recovery in the acute and chronic stages after SCI based on regression analyses [Furlan JC, Fehlings MG. J Neurotrauma. 2009;26(10):1707-17]. (2) Gender was not explored because of the above-mentioned low prevalence of spinal cord injuries among females.
Recruitment	We recruited individuals who had a cervical spinal cord injury. The inclusion criteria were: (1) single event traumatic or ischemic para- or tetraplegia, (2) first assessment possible within the first 4 weeks after incidence, (3) patient capable and willing of giving informed consent. This study was not susceptible to self-selection bias.
Ethics oversight	The research followed the Declaration of Helsinki and was approved by the Institutional Review Board of the following institutions: Bayrische Landesärztekammer, Ethik-Kommission (REB #188/2003; Bayreuth, Germany), Ethik-Kommission der Bayerischen Landesärztekammer (REB approval was waived because the project was treated as a data registry, but informed consent was obtained from all participants; Murnau, Germany), Universität Ulm Ethikkommission (REB #71/2005; Ulm, Germany), Universität Heidelberg Ethikkommission der Med. Fakultät (REB #S-188/2003; Heidelberg, Germany), Kanton Zürich Kantonale Ethikkommission (REB #EK-03/2004/PB_2016-00293; Zurich, Switzerland).

Note that full information on the approval of the study protocol must also be provided in the manuscript.

Field-specific reporting

Please select the one below that is the best fit for your research. If you are not sure, read the appropriate sections before making your selection.

☒ Life sciences ☐ Behavioural & social sciences ☐ Ecological, evolutionary & environmental sciences

For a reference copy of the document with all sections, see nature.com/documents/nr-reporting-summary-flat.pdf

Life sciences study design

All studies must disclose on these points even when the disclosure is negative.

Sample size	This study conducted a retrospective analysis of data from the European Multicenter Study about Spinal Cord Injury (EMSCI; ClinicalTrials.gov Identifier: NCT01571531) investigating the natural recovery after SCI. At the time of inquiry to the EMSCI (November 25th, 2020), the database included 5794 individuals with SCI - who were assessed between 2004-2020. Currently, the sample size of the EMSCI study is above the estimated sample size of 5500 individuals (ClinicalTrials.gov Identifier: NCT01571531). In the cohort of individuals described in this study, we based our sample size estimation on previous studies detecting the effects of natural recovery in SCI at the muscle level [167 individuals in Ditunno JF. Arch Phys Med Rehabil. 2000;81(4):389-393] and describing the proportional recovery rule in stroke [41 individuals in Prabhakaran S. Neurorehabil Neural Repair. 2008;22(1):64-71] [93 individuals in Byblow WD. Ann Neurol. 2015;78(6):848-859] [385 individuals in Bonkhoff AK. Brain. 2020;143(7):2189-2206]. The sample size utilized in the present study (748 individuals) is, thus, above the sample size used by similar studies in the past, which were able to detect the expected effects of natural or spontaneous recovery. Furthermore, this is the first study to assess the role of CST integrity for the proportional recovery after SCI - analyzing MEPs in a sample of 203 individuals [also above the 93 individuals utilized in Byblow WD. Ann Neurol. 2015;78(6):848-859 to describe a similar effect in post-stroke individuals].

Data exclusions	We excluded data from participants who did not fit our inclusion criteria. During the data curation process, further exclusions were performed of participants that did not have a complete dataset, to avoid the mean imputation of missing data. During our complex analysis (machine learning) we excluded muscles with the maximum muscle motor score at baseline to avoid ceiling effects in our predictions. All data exclusion is described in detail in our manuscript.
Replication	We extensively reproduced the findings reported in the manuscript. We utilized more than one statistical approach to describe the recovery profiles (Figure 3 and Supplementary Figures 2-5). Also, the data described in Figure 3 utilized a smaller sample of individuals who had very acute assessments (within the first week after the lesion). These findings are replicated in Supplementary Figures 2-5 employing a larger dataset of individuals who had assessments in the acute phase after the lesion (within the 4-weeks after the lesion). We also extensively replicated the outcomes of our machine learning models using binary logistic regressions, obtaining similar results (data not shown).
Randomization	There was no randomization of participants because the study included individuals who had a spinal cord injury and fitted our inclusion criteria. The effects of covariates were explored in our post-hoc analysis and prediction models.
Blinding	Data blinding was not relevant to the study because of the investigation of the natural recovery after a spinal cord injury utilizing a retrospective analysis of data from the European Multicenter Study about Spinal Cord Injury (EMSCI). Standard blinding procedures were conducted at the different centers participating on the EMSCI study - following the protocols in place at each site.

Reporting for specific materials, systems and methods

We require information from authors about some types of materials, experimental systems and methods used in many studies. Here, indicate whether each material, system or method listed is relevant to your study. If you are not sure if a list item applies to your research, read the appropriate section before selecting a response.

Materials & experimental systems

n/a	Involved in the study
☒	☐ Antibodies
☒	☐ Eukaryotic cell lines
☒	☐ Palaeontology and archaeology
☒	☐ Animals and other organisms
☐	☒ Clinical data
☒	☐ Dual use research of concern

Methods

n/a	Involved in the study
☒	☐ ChIP-seq
☒	☐ Flow cytometry
☒	☐ MRI-based neuroimaging

Clinical data

Policy information about clinical studies

All manuscripts should comply with the ICMJEguidelines for publication of clinical research and a completedCONSORT checklist must be included with all submissions.

Clinical trial registration	NCT01571531
Study protocol	https://www.clinicaltrials.gov/ct2/show/NCT01571531
Data collection	Data collection occurred in dedicated spinal cord injury centers: the Hohe Warte Bayreuth (Bayreuth, Germany), BG-Trauma Center (Murnau, Germany), RKU Universitäts- und Rehabilitationskliniken Ulm (Ulm, Germany), Spinal Cord Injury Center of Heidelberg University Hospital (Heidelberg, Germany), and Spinal Cord Injury Center - Balgrist University Hospital (Zurich, Switzerland). Data recruitment and collection occurred between 2004-2020.
Outcomes	The primary outcome was the strength recovery in the first year after the spinal cord injury, defined a priori at the planning stage of the study. The strength recovery was assessed both for the upper extremity and for the individuals as a whole (sum of the upper and lower extremity strength). The strength recovery profile was assessed by our clinical teams utilizing the manual muscle testing score system (scores from 0-5 for each myotome; it sums up to 50 points for the upper extremity and up to 100 points for the individual). The secondary outcomes were baseline covariates: ASIA classification, distance from the motor level, strength and sensation, and electrophysiological multimodal assessments. These outcomes are assessed by the International Standards for Neurological Classification of Spinal Cord Injury (ISNCSCI), except the electrophysiological multimodal assessments - which were assessed following standard procedures described in the Neurophysiology Manual used within EMSCI (www.emsci.org).
